# ShapeEditor: A StyleGAN Encoder for Stable and High Fidelity Face Swapping

**DOI:** 10.3389/fnbot.2021.785808

**Published:** 2022-01-21

**Authors:** Shuai Yang, Kai Qiao, Ruoxi Qin, Pengfei Xie, Shuhao Shi, Ningning Liang, Linyuan Wang, Jian Chen, Guoen Hu, Bin Yan

**Affiliations:** Henan Key Laboratory of Imaging and Intelligent Processing, People's Liberation Army (PLA) Strategy Support Force Information Engineering University, Zhengzhou, China

**Keywords:** face swapping, generative adversarial network, disentanglement, style transfer, deepfake

## Abstract

With the continuous development of deep-learning technology, ever more advanced face-swapping methods are being proposed. Recently, face-swapping methods based on generative adversarial networks (GANs) have realized many-to-many face exchanges with few samples, which advances the development of this field. However, the images generated by previous GAN-based methods often show instability. The fundamental reason is that the GAN in these frameworks is difficult to converge to the distribution of face space in training completely. To solve this problem, we propose a novel face-swapping method based on pretrained StyleGAN generator with a stronger ability of high-quality face image generation. The critical issue is how to control StyleGAN to generate swapped images accurately. We design the control strategy of the generator based on the idea of encoding and decoding and propose an encoder called ShapeEditor to complete this task. ShapeEditor is a two-step encoder used to generate a set of coding vectors that integrate the identity and attribute of the input faces. In the first step, we extract the identity vector of the source image and the attribute vector of the target image; in the second step, we map the concatenation of the identity vector and attribute vector onto the potential internal space of StyleGAN. Extensive experiments on the test dataset show that the results of the proposed method are not only superior in clarity and authenticity than other state-of-the-art methods but also sufficiently integrate identity and attribute.

## 1. Introduction

As one of the main contents of deepfake, face swapping declares to the world today that seeing is not always believing. Face swapping refers to transferring the identity of a source image to the face of another target image while keeping unchanged the illumination, head posture, expression, dress, background, and other attribute information of the target image. Face swapping has received widespread attention since its birth, catering to the affluent needs of social life, such as hairstyle simulation, film and television shooting, privacy protection, and so on (Ross and Othman, 2010).

Face swapping is accompanied not only by its interesting and operational application prospects but also by various challenges between reality and vision. The early face-swapping methods (Bitouk et al., 2008; Korshunova et al., 2017) require many images of source and target characters to provide sufficient facial information. Otherwise, the models would not have a suitable reference basis to produce good results. Some three-dimensional-based (3D-based) methods (Olszewski et al., 2017; Nirkin et al., 2018; Sun et al., 2018) make use of the advantage of fitting 3D face models to deal with the problems of large angle and small samples. At the same time, due to the limited accuracy of 3D face models, it is impossible to generate works with better details and higher fidelity. Recently, with the continuous tapping of the potential of generative adversarial networks (GANs) (Nandhini Abirami et al., 2021), some face-swapping methods based on GANs (Bao et al., 2018; Natsume et al., 2018a,b; Li et al., 2019; Nirkin et al., 2019) can achieve a good fusion of identity and attribute information with only a small number of samples, reflecting the effect of great creativity. Unfortunately, the surprising creativity of these methods does not offset the adverse impacts of their frequent artifacts and low-resolution limitation.

On another track, the most advanced face image generation methods have generated facial images with high resolution and realistic texture. Most notably, StyleGAN (Karras et al., 2019) can randomly generate a variety of clear faces with a resolution of up to 1024 × 1024. StyleGAN has three potential spaces: initial potential space Z, intermediate potential space W, and extended potential space W+. (Abdal et al., 2019) proved that the concatenation of 18 different 512-dimensional vectors is the easiest way to embed an image and obtain a reasonable result. On this basis, various works (Gu et al., 2020; Härkönen et al., 2020; Richardson et al., 2020; Zhu et al., 2020) explore in detail the StyleGAN potential vector space: some (Shen and Zhou, 2020; Shen et al., 2020; Tewari et al., 2020) find a linear direction to control the change of a single facial attribute, some (Nitzan et al., 2020) control facial expression and posture in the original StyleGAN image domain, and others (Richardson et al., 2020; Wang et al., 2021) deal well with the difficult task of facial super-resolution.

In contrast with other face-swapping methods, the first criterion we pursue is that the images after face swapping have both higher clarity and better authenticity. We propose a many-to-many face-swapping method based on the pretrained StyleGAN model (Karras et al., 2019), which strives to ensure the clarity and fidelity of the results while fusing identity and attribute information. Given the inherent ability of the pretrained StyleGAN model to generate random high-quality face images, the difficulty of this task is how to accurately render the corresponding latent vectors. To achieve this goal, we first designed an encoder, ShapeEditor, to find the corresponding codes in the W+ vector space. The workflow of the encoder was divided into two stages, the first being the respective extraction of identity and attribute codes, and the second being to map the combination of two-channel codes into the potential input vector domain of the pretrained model. Moreover, we designed a set of loss functions with a strong monitoring ability to urge ShapeEditor to update parameters to learn to map step by step onto the latent space of StyleGAN. As verification, we made numerous qualitative and quantitative experimental comparisons with the existing face-swapping methods, which show the unique advantages of the proposed method.

## 2. Related Works

Recently, the GAN-based face-swapping methods have shown better performance, thus attracting more extensive research and attention. Although integrate attributes and identity information well, these methods generally have the common problem of poor clarity and authenticity. On the other hand, as GAN with better image quality has been proposed, many works are devoted to manipulating GAN's semantic space to generate clear and stable images. We creatively combine the advantages of the above two fields to improve the performance of face swapping, and make possible the more complex control of GAN's potential space.

### 2.1. GAN-Based Face Swapping

**Figure 1:**
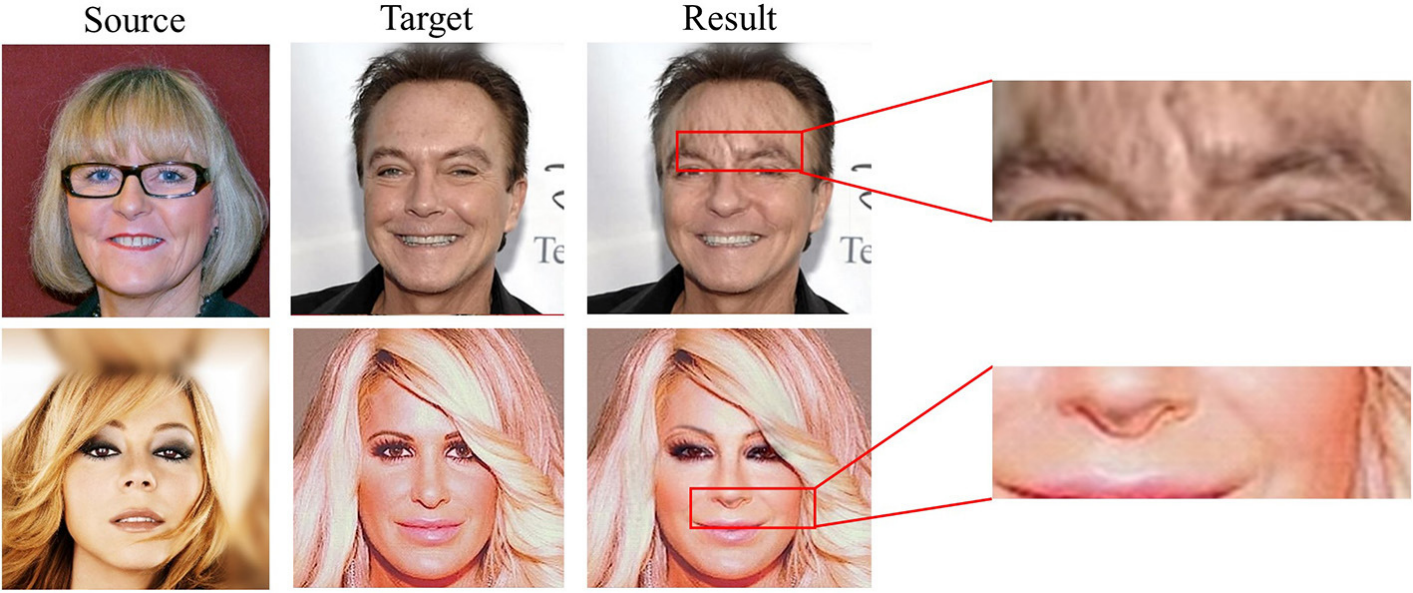
Some abnormal results generated by FaceShifter (Li et al., 2019).

Olszewski et al. (2017) fit the 3D face model of the source face and used a conditional generator of the coder-decoder structure to infer the converted face texture. Too simple generator network structure and training strategy make this method unable to separate identity and attribute information to further complete many-to-many identity exchange. Sun et al. (2018) trained a convolutional neural network to regress the parameters of a 3D model of the input face, replaced the identity parameters, and combined the region around the head to generate a realistic face-swapped image. Limited to the accuracy of the model reconstruction, 3D-based face-swapping methods are unsatisfactory in terms of attribute and identity fidelity. Face Swapping GAN (FSGAN) (Nirkin et al., 2019) used sparse landmarks to track facial expression, and designed GANs with different functions for the three stages of face swapping. This method realized subject agnostic face swapping, while being limited by the resolution of the input image and the complexity of expression. Bao et al. (2018) implemented this task using a more concise coder-decoder architecture, in which two independent coders separate the identity and attributes of human faces. This method used an asymmetric training strategy to promote a large number of unlabeled faces to contribute to the training. Following the basic network framework and asymmetric training strategy of Bao et al. (2018), FaceShifter (Li et al., 2019) has done meaningful work on embedding multi-level information in the generator and handling occlusion more robustly. The generator leverages denormalizations for feature integration in multiple feature levels, showing a better representation of identity and attribute. However, the clarity and stability of the image generated by FaceShifter are not always ideal. As shown in Figure 1, the eyebrows of the result in the first line appear ghosting, and the nose of the result in the second line appear artifact. These examples show that the most advanced GAN-based face-swapping method is still insufficient in authenticity.

### 2.2. The Potential and Challenge of Pretrained GAN Manipulation

While a lot of works have been done on how to control GAN to perform complex image operations, such as face swapping, others focus on improving the quality of images. Through carefully designed style-based network structure and layer-by-layer training, StyleGAN (Karras et al., 2019) realized high-definition and high-quality face image generation. With the help of pretrained StyleGAN, image quality is easier to be improved. The manipulation of StyleGAN is a difficult task, and most early works are limited to understanding and reproducing the potential space of GAN. The inversion task of StyleGAN is to find the potential vector that best matches the given image. Abdal et al. (2019) took several minutes to embed a face into the StyleGAN image domain. Richardson et al. (2020), Zhu et al. (2020), and Gu et al. (2020) tried to improve efficiency using encoder structure, but the inversion results of wild images in their methods are unsatisfactory. Later, some more complex works appeared, such as changing individual attributes (smile, age, facial angle, etc.) (Härkönen et al., 2020; Shen and Zhou, 2020; Shen et al., 2020), establishing relationship between 3D semantic parameters and genuine facial expressions (Tewari et al., 2020), and super-resolution of low-quality facial images (Wang et al., 2021). To the best of our knowledge, there is no face-swapping method based on StyleGAN. This task requires more complex semantic manipulation, and the current controllers are not competent. Nitzan et al. (2020) did closely related work to control expression through latent space mapping. However, working in the W space led to the failure of embedding wild images into potential space. In addition, the single vector of the attribute is too plain to carry the information of background, posture, expression, etc.

### 2.3. The Inheritance and Transcendence

We propose a StyleGAN encoder, called ShapeEditor, for stable and high-fidelity face swapping. As the combination of face swapping and pretrained GAN manipulation, ShapeEditor inherits and surpasses the latest ideas in the two fields.

We use an asymmetric training strategy similar to that in FaceShifter (Li et al., 2019) to realize the training process without labeled data, so as to ensure solid constraints and reduce data processing costs. Moreover, the well-designed coder-decoder structure of our framework can firmly guarantee image quality, which is the weakest aspect of FaceShifter. Inspired by SPADE (Park et al., 2019) and AdaIN (Huang and Belongie, 2017), the FaceShifter generator designs AAD layer-level denormalization for feature integration in multiple feature levels. By comparison, the internal mapper of ShapeEditor is composed of lightweight Multilayer Perceptrons (MLP) to generate feature vectors embedded in StyleGAN W+ space, which reduces the burden of model training.

Our method and Nitzan et al. (2020) both use the decoupling framework to extract attribute and identity code through attribute extractor and identity extractor, respectively. The codes are then mapped into the latent space of the employed pretrained generator. Our key difference is that we select W+ potential space as the mapping space, which is the premise of realizing the complex semantic operation of face swapping. In addition, in order to recover the attribute information more finely, we use multi-level feature mapping instead of a single output as attribute code like Nitzan et al. (2020) did. The ablation study proves that our pertinent designs make a significant contribution to better semantic manipulation.

## 3. Methods

**Figure 2:**
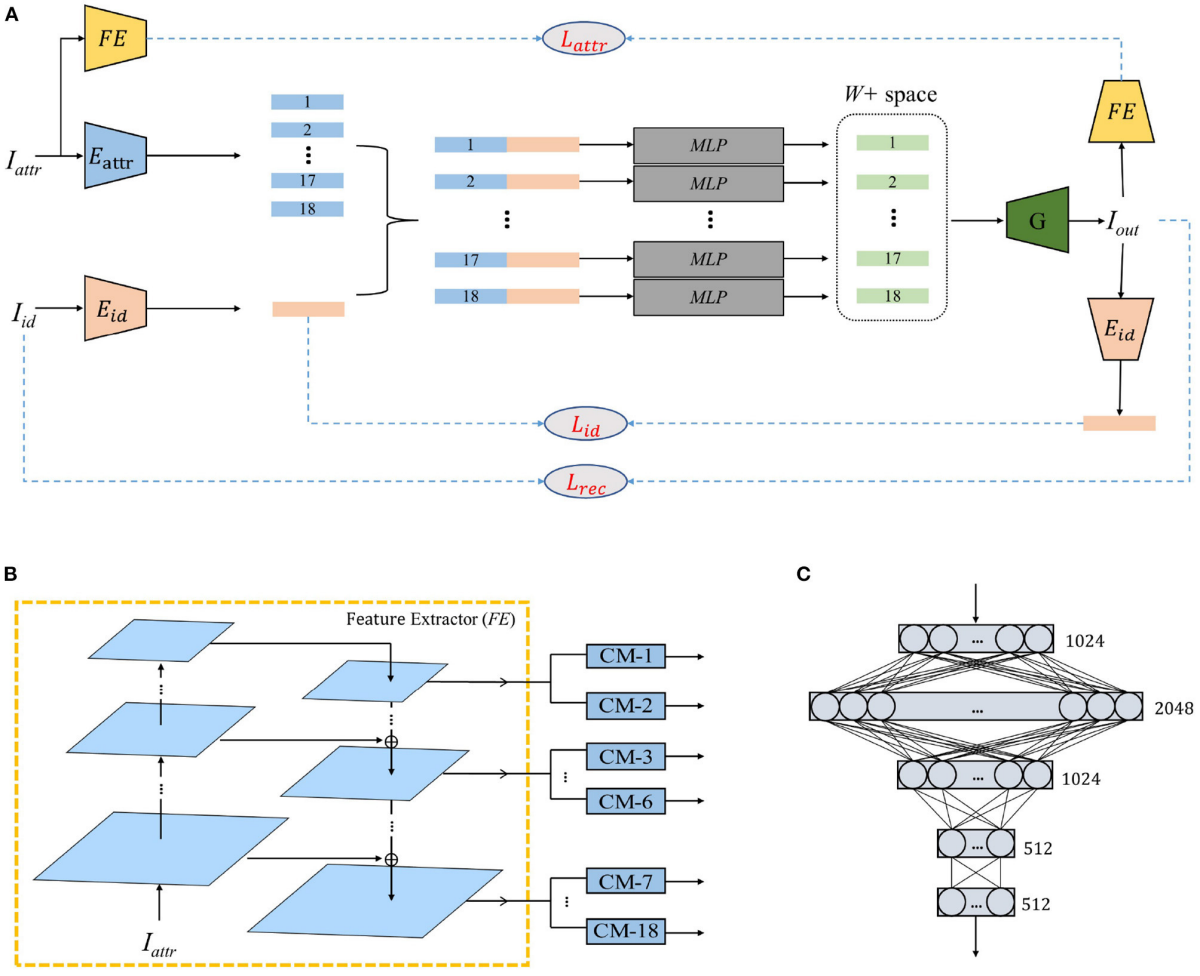
The overall structure and data flow of the proposed model. **(A)** is the flow of our method. **(B)** is the structure of *E*_attr_. **(C)** is the structure of Multilayer Perceptron (MLP).

Our method requires two images as input: *I*_attr_ and *I*_id_. We expect the output of the model to reflect the identity of *I*_id_ and the facial expression, head posture, hairstyle, lighting, and other attribute information of *I*_attr_. Therefore, the main challenge of this work is to obtain the StyleGAN potential vectors that are consistent with the W+ spatial distribution and better integrate attributes and identity. To solve this problem, we designed a two-step coding process. As shown in Figure 2A, the entire mapping process is divided into two phases: ID-ATTR encoding and latent-space encoding. In the first stage, *E*_id_ extracts the identity vector of *I*_id_, and *E*_attr_ extracts the attribute vector of *I*_attr_. As shown in Figure 2B, inspired by pSp (Richardson et al., 2020), *E*_attr_ consists of a pyramid-shaped three-layer feature map extraction structure and a set of convolutional mappers (CM). In the second stage, we input the concatenation of *E*_id_(*I*_id_) and *E*_attr_(*I*_attr_) into the multilayer perceptron (MLP) of each layer and map the vectors containing identity and attribute information directly to the W+ potential vector space. In summary, the whole image conversion process can be represented as


(1) $$\begin{array}{l} I_{\text{out}}=G(MLP(\left[E_{\text{id}}\left(I_{\text{id}}\right),E_{\text{attr}}\left(I_{\text{attr}}\right)])\right), \end{array}$$

where *G*(·) is the pretrained StyleGAN model, *MLP*(·) is the multilayer perceptron, and [·, ·] is the concatenation of two vectors.

### 3.1. Network Architecture

*E*_id_ is pretrained ArcFace (Deng et al., 2019) model. We use ResNet-IR (Deng et al., 2019) for Feature Extractor (*FE*), in which the feature output layers are 27, 30, and 44. The CM is a fully convolutional network that compresses the tensor of 8 × 8 × 512 dimensions into 1 × 1 × 512 dimensions through three convolution operations with a step size of two. As shown in Figure 2C, *MLP* is a five-layer fully connected network. The StyleGAN generator is a pretrained model trained on FlickrFaces-HQ (FFHQ) (Karras et al., 2019).

We mainly use convolution to reduce the dimensions of image encoding and use deconvolution to decode W+ vectors. *E*_attr_ and *E*_id_ achieve the data-dimension reduction from image to vector through convolution and other network operations. The identity vector and attribute vector dimensions are both 1 × 512. The splicing of identity and attribute vectors is then input into a set of MLP to convert the face style and map the low-dimensional information to W+ space. The deconvolution process is mainly reflected in StyleGAN, which changes from vectors in W+ space to images. Note that we do not change any structure of StyleGAN but hope to use its powerful image-generation capabilities to make our face-changing images more stable and clear.

### 3.2. Training and Loss Functions

The advanced face-recognition model accurately identifies the face, so we believe that it can extract face-feature information and take the feature vector extracted by the pretrained ArcFace (Deng et al., 2019) as the identity information. To ensure that the identity of *I*_out_ is consistent with *I*_id_, we introduce the identity loss


(2) $$\begin{array}{l} L_{\text{id}}=∥E_{\text{id}}\left(I_{\text{id}}\right)-E_{\text{id}}\left(I_{\text{out}}\right){∥}_{2}, \end{array}$$

where *E*_id_(·) is the pretrained ArcFace model.

Similarly, we adopt certain restrictions to ensure that the attribute information of *I*_out_ is consistent with that of *I*_attr_. Given that the three-layer feature map extraction structure should gradually have the ability to extract attribute information with the training process, we define the attribute loss function as


(3) $$\begin{array}{l} L_{\text{attr}}=∥P\left(I_{\text{attr}}\right)-P\left(I_{\text{out}}\right){∥}_{2}^{2}, \end{array}$$

where *P*(·) is the extraction structure.

Note that the attribute information of *I*_attr_ and the identity information of *I*_id_ should not only exist in *I*_out_ but should also be well integrated. Based on this idea, we define the reconstruction loss as


(4) $$\begin{array}{l} L_{\text{rec}}=\{\begin{array}{ll} ∥I_{\text{out}}-I_{\text{id}}{∥}_{2}+∥F\left(I_{\text{out}}\right)-F\left(I_{\text{id}}\right){∥}_{2} & \text{if }I_{\text{id}}=I_{\text{attr}}\\ 0 & \text{otherwise}, \end{array} \end{array}$$

where *F*(·) is the perceptual feature extractor in the loss of learned perceptual image patch similarity (Zhang et al., 2018), which extracts the perceptual information of the image at the high-dimensional level. $L_{2}$ loss measures the difference between the two images at the pixel level. Note that $L_{\text{rec}}$ has a positive value only when *I*_id_ and *I*_attr_ are the same because only in this case should *I*_out_ and *I*_id_ (or *I*_attr_) be so consistent that they are exactly the same; otherwise, we cannot expect a similar comparison between the two images. Overall, our total training loss is the weighted sum of all the losses mentioned above:


(5) $$\begin{array}{l} L_{\text{total}}=\lambda_{\text{id}}L_{\text{id}}+\lambda_{\text{attr}}L_{\text{attr}}+\lambda_{\text{rec}}L_{\text{rec}}. \end{array}$$

Based on the loss functions and model structure proposed above, we train the ShapeEditor encoder according to Algorithm 1.

**Algorithm 1:** Training ShapeEditor using gradient descent.

**Input:** ***I***_attr_: Image containing attribute information ***I***_id_: Image containing identity information *P*: Identity-attribute image pair space **Functions:** **Encoder ShapeEditor**:*P* →W+ **Generator G**:W+→I **Loss** ← $L_{\text{id}}:$ Calculate the identity loss between *I*_id_ and *I*_out_. **Loss** ← $L_{\text{attr}}:$ Calculate the attribute loss between *I*_attr_ and *I*_out_. **Loss** ← $L_{\text{rec}}:$ Calculate the reconstruction loss between *I*_id_(*I*_attr_) and *I*_out_. **Output:** *I*: Image space W+: Potential vector space of StyleGAN *I*_out_: Synthesized face-swapping image
1: **for** number of training iterations **do:**
2: **for** *I*_id_, *I*_attr_ randomly selected in training dataset **do:**
3: Generate the W+ space vector using [*I*_id_, *I*_attr_]
4: ShapeEditor:*P* →W+
5: Generate the face-swapping image *I*_out_ using the W+ space vector
6: G:W+→I
7: Calculate the identity loss $L_{\text{id}}$, the attribute loss $L_{\text{attr}}$, and the reconstruction loss $L_{\text{rec}}$
8: Update ShapeEditor with loss
9: end
10: end.

## 4. Experiments

**Implementation Details:** We use the FFHQ (Karras et al., 2019) dataset as the training set, and the value of loss weights is set to λ_id_ = 0.5, λ_attr_ = 0.1, λ_rec_ = 1. The ratio of the training data with *I*_id_ = *I*_attr_ to that with *I*_id_≠*I*_attr_ is set to 2:1. During the training, the network parameters of *E*_id_ and the StyleGAN generator remain unchanged, and the weights of the rest are updated with iterations. To compare with other methods, we train the model with images of 256 × 256 resolution in this section. This model was trained on a single NVIDIA TITAN RTX for about 2 days with a Ranger optimizer (Richardson et al., 2020), with a batch size set to eight and a learning rate set to 0.0001.

### 4.1. Qualitative Comparison With Previous Methods

**Figure 3:**
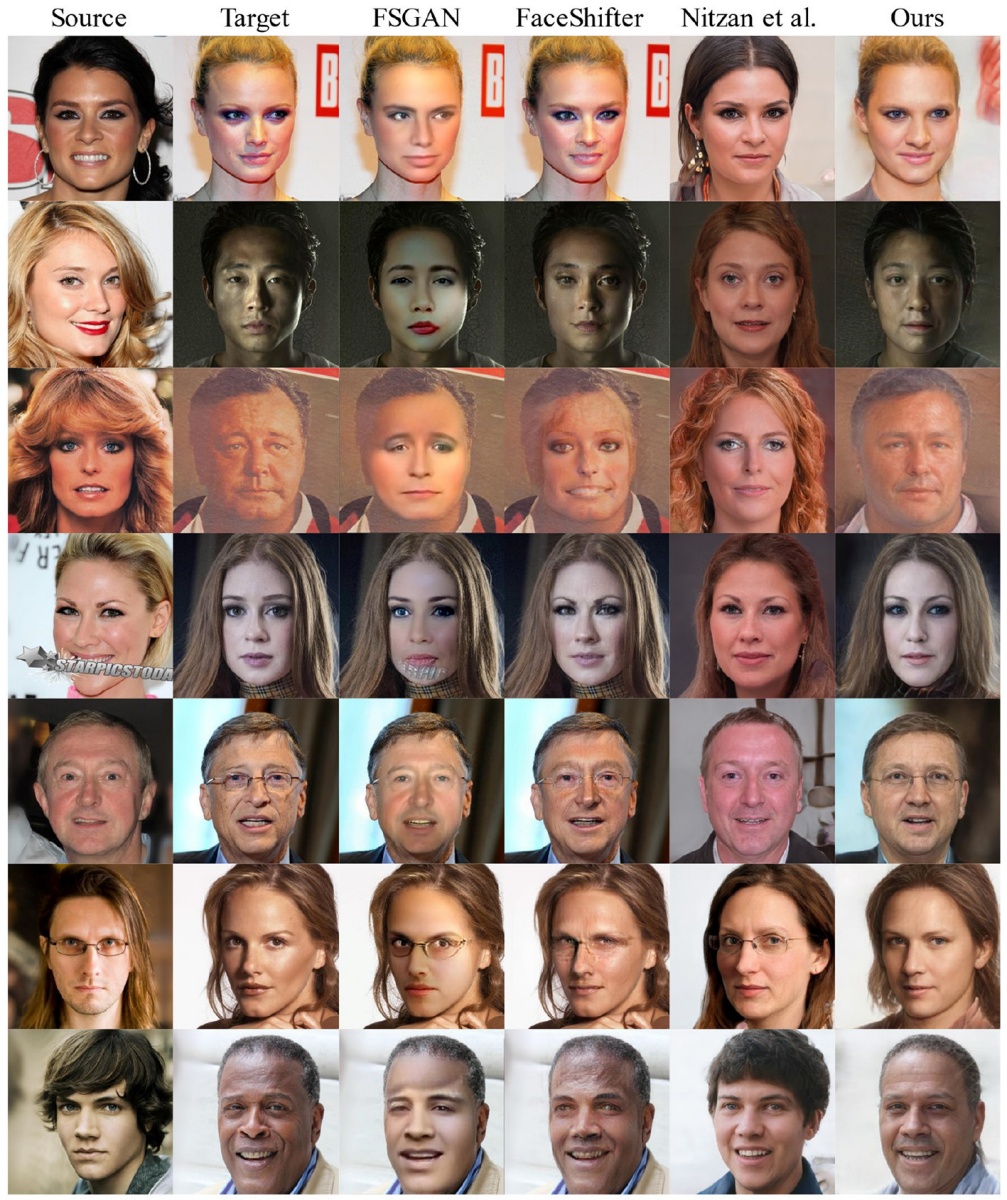
Qualitative comparison with FSGAN (Nirkin et al., 2019), FaceShifter (Li et al., 2019; Nitzan et al., 2020) on the CelebAMask-HQ (Lee et al., 2020) test dataset.

We compare the proposed method with FSGAN (Nirkin et al., 2019), FaceShifter (Li et al., 2019; Nitzan et al., 2020) on the CelebAMask-HQ (Lee et al., 2020) test dataset. Figure 3 shows, as expected because the proposed method is based on a pretrained StyleGAN (Karras et al., 2019) with high-quality face-generation capabilities, that all the generation results (Figure 3, column 6) are stable and clear enough that there are no errors such as artifacts and abnormal illumination.

Almost every output image (Figure 3, column 3) of FSGAN (Nirkin et al., 2019) shows unnatural lighting transition and lack of facial details, the abnormal region of the face is caused by directly extracting and filling the internal area of the face (Figure 3, row 3, column 4), which is completely avoided in the proposed method.

Because there is no pretrained model as the backbone, it is difficult for FaceShifter (Li et al., 2019) to avoid facial blur, some results even show facial illumination confusion (Figure 3, row 3, column 4) and eye ghosting (Figure 3, row 7, column 4), showing that its authenticity is significantly inferior to that of the proposed method.

Similar to the proposed method, Nitzan et al. (2020) use StyleGAN (Karras et al., 2019) as the backbone. However, it cannot accurately integrate identity and attribute information because of its simple encoder structure and the constraint of W potential space. Therefore, although it can generate high-quality images (Figure 3, column 6), it is not as good as the proposed method for fusing semantic information, which is reflected in the attributes of the target image, such as hairstyle and background, that are not contained.

In addition to the excellent performance in terms of authenticity and fidelity, the proposed method also deals with extreme lighting conditions (Figure 3, row 2, column 6) and even keeps the sense of age (Figure 3, row 3, column 6). Thanks to that, we use the facial recognition module to extract the identity vector instead of directly using the pixels in the facial area. We can extract the identity information very well even if the source image has facial occlusion (Figure 3, row 4, column 6). The proposed model understands whether its output should have glasses (Figure 3, column 6, rows 5 and 6), which is embedded in the potential space of the pretrained StyleGAN model (Karras et al., 2019).

### 4.2. Quantitative Comparison With Previous Methods

As mentioned in the section 2.3, our method mainly inherits the ideas of latent space manipulation of pretrained models and GAN-based face swapping. To show the advantages, we compare the proposed method with other related. In the field of latent space manipulation, Nitzan et al. (2020) is the most similar to our work, which is about controlling facial attributes with StyleGAN. In the field of GAN-based face swapping, DeepFakes (Rössler et al., 2019), FSGAN (Nirkin et al., 2019), and FaceShifter (Li et al., 2019) occupy earlier positions and have achieved remarkable face exchange. To show the robustness of our method, we compare the proposed method with them quantitatively.

#### 4.2.1. Comparison With Nitzan et al.

Our method and Nitzan et al. (2020) both make use of the image generation ability of pretrained StyleGAN, and make efforts to achieve adequate control of the human face. But we are different in the choice of mapping space and framework design. To show the significance of our improvement in semantic control, we quantitatively compare our method with Nitzan et al. (2020) in terms of identity, pose, expression, and mood consistency on CelebAMask-HQ (Lee et al., 2020) dataset.

The face swapping model not only needs to ensure the image quality but also needs to fuse the identity and attribute information to the greatest extent. We propose four indicators to measure these aspects. To calculate the identity information in the test stage, we use another advanced method called CurricularFace (Huang et al., 2020) as the face-recognition module to extract the identity vectors of source faces and face-swapping results, then use L2 distance to calculate the difference between them to get the identity error. To ensure that the conversion results are consistent with the target image in attribute, we use 3DDFA-V2 (Guo et al., 2020) to estimate the key face points and the head angle. For normalization, we use the two-dimensional (2D) coordinate information instead of 3D coordinate information to reduce the error impact of key-point estimation as much as possible, and calculate the average position of key points in each image, and then obtain the relative position of each point so as to establish a unified expression coordinate system. Based on the above, we take the difference between the target image and the resulting image in angle as pose error, in key face points as expression error. In addition to pose and expression, mood embodies the high-level semantics of face attribute. Inspired by Abirami and Vincent (2021), we use the emotion recognition model (Zhao et al., 2021) to detect the ability of face-swapping methods to transmit emotional information. Specifically, we recognize the moods of the swapped images and calculate the consistency of the mood recognition results before and after face exchange.

We randomly extract images from the CelebAMask-HQ dataset as source faces and take the remaining images as target faces to form one-to-one corresponding face combinations as the test dataset. As shown in Table 1, our method is superior to Nitzan et al. (2020) in pose error, expression error, and mood consistency, which shows our advantages in attribute information transfer. Our identity error is slightly higher than Nitzan, that is because face swapping brings more changes in head area than expression manipulation. Our advantages in most indicators demonstrate that we have realized better work in latent space manipulation.

**Table 1:** Quantitative comparison with Nitzan et al. (2020). Our method performs better in most indicators.

**Method**	**Identity Error** **↓**		**Pose Error** **↓**		**Expression Error** **↓**		**Mood Consistency ↑**
	**Avg**.	**Std**.	**Avg**.	**Std**.	**Avg**.	**Std**.	**Acc. (%)**
Nitzan et al. (2020)	**0.97**	0.30	5.99	7.16	10.13	5.38	65.35
Ours	1.30	0.33	**3.82**	6.88	**5.93**	3.63	**75.38**

*Bold values represent the best. ↑ represents that the larger the value, the better. ↓ represents that the smaller the value, the better*.

#### 4.2.2. Comparison With Face Swapping Methods

To comprehensively show the face-swapping ability of our method, we conduct quantitative comparisons in transformation consistency and image quality with DeepFakes, FSGAN, and FaceShifter. Our work, FSGAN, and FaceShifter rely on a single reference or few references and are many-to-many approaches. At the same time, DeepFakes have to be supported by multi-images or videos to transfer faces in to two specific identities. Therefore, in order to ensure the effectiveness and efficiency of comparison, we extract DeepFakes conversion results from Rössler et al. (2019) dataset. The calculations of identity error, pose error, expression error, and mood consistency is the same as in section 4.2.1, which represent transformation consistency evaluation. Following the work of Yao et al. (2020), we employ peak signal-to-noise ratio (PSNR) (Huynh-Thu and Ghanbari, 2008) and structural similarity index (SSIM) (Wang et al., 2004) to measure the image reconstruction similarity between the target face and swapped face. Last but not least, to evaluate the clarity and authenticity of images, we use Li and Lyu (2018), which can effectively capture the artifacts in the forged images, to identify fake faces according to the resolution of the generated images. Specifically, we calculate the Forgery Detection Rate (FDR) of the output images. In the analysis of section 4.1, we know that the problems of low-quality images are mainly reflected in insufficient resolution and abnormal artifact areas. Therefore, the method of Li and Lyu (2018) can evaluate the quality of face images to a certain extent.

Table 2 lists the comparison results of different face-swapping methods. Notably, our method performs best in SSIM, indicating that our method retains the brightness, contrast, and structure of the original images to the greatest extent. Besides, our method outperforms others in PSNR, which demonstrates that our method can better preserve the global similarity than others. Also, our method has the least scores in FDR under different thresholds, which implies that our method can generate images with more sufficient resolution and less abnormal artifact areas. Finally, it is worth noting that our method has the second-best or the same level scores in identity error, pose error, expression error, and mood consistency, indicating that our method is comparable to others in identity and attribute, while being superior to them in terms of image quality and stability.

**Table 2:** Quantitative assessment with DeepFakes (Rössler et al., 2019), FSGAN (Nirkin et al., 2019), and FaceShifter (Li et al., 2019).

		**DeepFakes**	**FSGAN**	**FaceShifter**	**Ours**
Identity Error ↓	Avg.	1.35	1.51	**0.96**	1.30
	Std.	0.32	0.45	0.31	0.33
Pose Error ↓	Avg.	3.79	**2.81**	3.04	3.82
	Std.	1.99	4.41	6.70	6.88
Expression Error ↓	Avg.	8.82	5.03	**4.53**	5.93
	Std.	3.30	2.17	2.83	3.63
Mood Consistency ↑	Acc. (%)	39.80	72.77	**77.94**	75.38
SSIM ↓	Avg.	0.81	0.95	0.96	**0.75**
	Std.	0.09	0.03	0.03	0.08
PSNR ↓	Avg.	20.54	23.76	28.17	**20.22**
	Std.	2.60	2.30	1.92	1.62
FDR ↓	Tsd.=0.01	91.42	76.59	37.67	**15.18**
	Tsd.=0.05	83.83	48.99	11.66	**2.67**
	Tsd.=0.1	77.45	35.86	6.05	**1.09**
	Tsd.=0.2	70.86	24.22	2.86	**0.32**

*Tsd. represents the threshold, which is set to judge whether samples are forged or not. Bold values represent the best. ↑ represents that the larger the value, the better. ↓ represents that the smaller the value, the better*.

### 4.3. Ablation Study

To verify the effectiveness of each component of the proposed method, we do the ablation study by evaluating the following degenerate models of our method:

- *Random StyleGAN*. Using randomly initialized StyleGAN instead of pretrained generator.
- *Single attribute vector*. This variant uses a single output layer of Feature Extractor (*FE*), while the original uses multi-layer attribute information.
- W *space*. Using W potential space instead of W+.
- *Random* *E*_id_. Using randomly initialized *E*_id_ instead of pretrained face recognition model, with weight updating.

**Figure 4:**
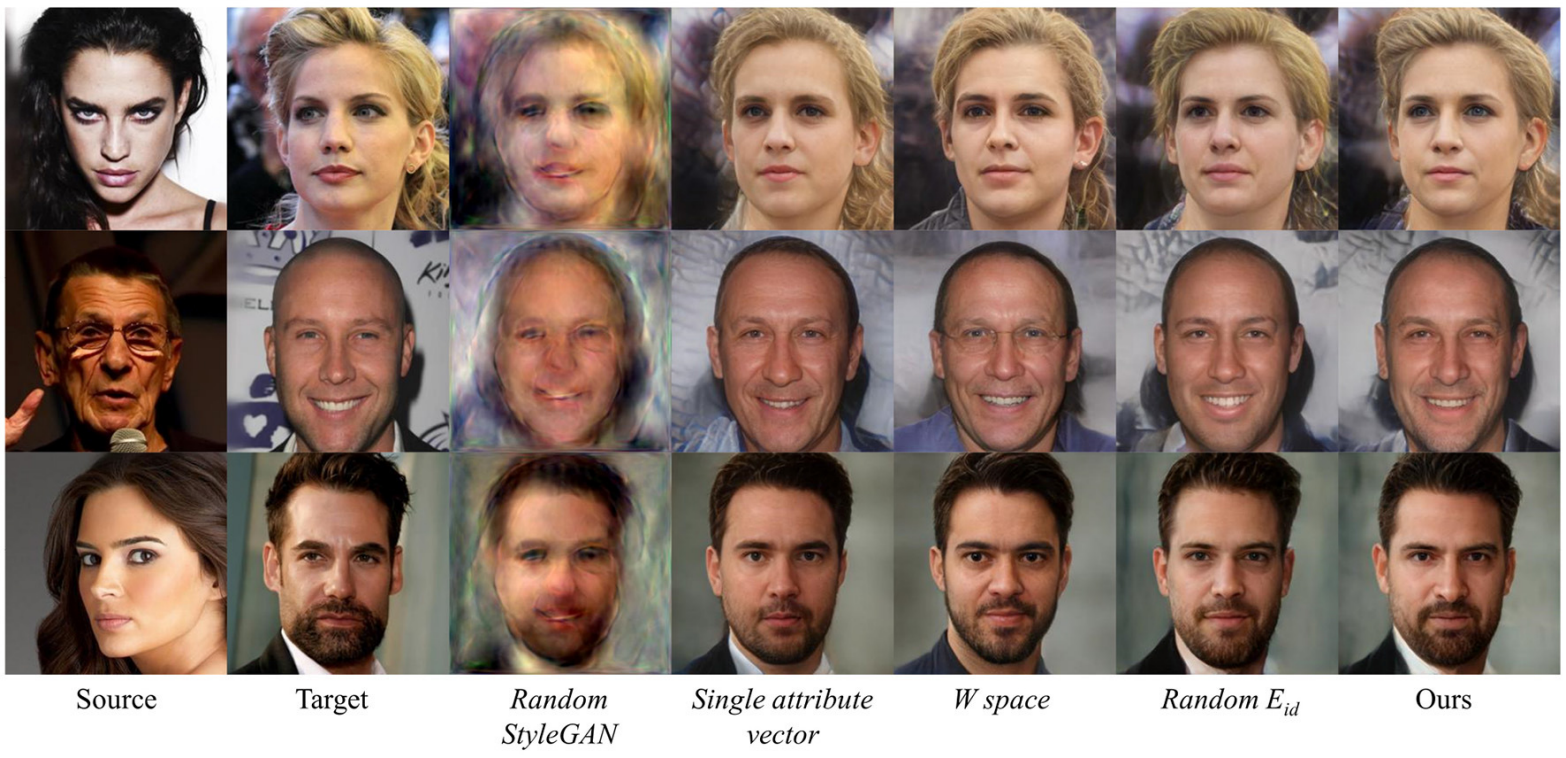
Qualitative ablation study on different variants. Our original model performs better than others.

We report the qualitative results of the variants of our method in Figure 4. We can see that our original model has better face-swapping results. The results of *Random StyleGAN* are too vague to recognize, indicating that the pretrained StyleGAN can help to generate clear and vivid faces. The results of *Single attribute vector* lose details of hair, wrinkles, and beard compared with ours, showing that multi-layer *FE* can deliver more attribute information. The results of W *space* leak identity information and add unnecessary details like glasses, showing that W+ potential space can more strictly embed wild faces into StyleGAN semantic space. The results of *Random* *E*_id_ leak identity information, which implies that using pretrained identity recognition model is of great significance.

Table 3 shows the quantitative results of the variants of our method on the randomly selected data from Lee et al. (2020) dataset. With the help of W+ space and pretrained *E*_id_, ours and *Single attribute vector* obtain lower identity error. The results of W *space* are much inferior compared to ours in pose error and expression error, revealing the importance of the reasonable space choice. Also, we can see that W *space* performs best in PSNR and SSIM, that is because face swapping in W space tends to map a wild face to a most similar face in the StyleGAN face domain, which is a more natural result with better image quality. Thanks to the help of StyleGAN, every model in Table 3 surpasses the existing face-swapping methods in PSNR and SSIM.

**Table 3:** Quantitative ablation study on different variants for face swapping.

		***Single attribute vector***	W ***space***	***Random E*_id_**	**Ours**
Identity Error ↓	Avg.	**1.29**	1.33	1.37	**1.29**
	Std.	0.32	0.33	0.34	0.33
Pose Error ↓	Avg.	3.94	4.53	4.05	**3.64**
	Std.	5.59	5.74	5.75	5.55
Expression Error ↓	Avg.	6.63	7.43	6.48	**5.96**
	Std.	3.45	4.20	3.87	3.22
PSNR ↓	Avg.	19.38	**18.42**	19.94	20.22
	Std.	1.51	1.58	1.59	1.62
SSIM ↓	Avg.	0.73	**0.70**	0.74	0.75
	Std.	0.08	0.09	0.07	0.08

*Bold values represent the best. ↓ represents that the smaller the value, the better*.

### 4.4. Discussion

**Figure 5:**
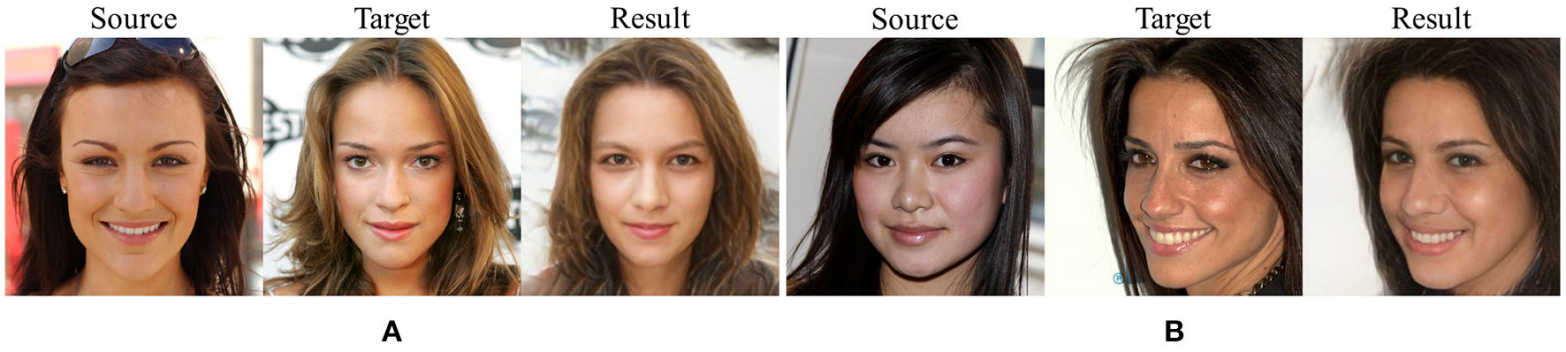
Shortcomings of the proposed model. The problem in panel **(A)** is that the background of the conversion result is blurred. The problem in panel **(B)** is that the swapped face lacks Asian characteristics.

The core of the proposed model is to use StyleGAN as the face decoder, which reduces the burden of face spatial feature learning and dramatically reduces the possibility of artifacts in the conversion results. However, the proposed method also has some defects. As shown in Figure 5A, the letters in the background of the target image become blurred in the resulting image, which shows that the proposed model is not good at restoring the background. Although the pretrained model we use learns the potential features of face space, it does not learn well how to separate the head from the background. To deal with this problem, we will separate the head and background in the next step through image segmentation and then combine the background of the target image with the head of the resulting image. At the same time, Figure 5B shows that the resulting image lacks Asian characteristics similar to those in the source image, which reflects the problem of insufficient potential vectors in the StyleGAN face space and is caused by the relative lack of Asian faces in the training dataset. Therefore, adding more types of faces to the pretrained model and selecting a better-pretrained model should also be a focus in future work.

## 5. Conclusion

This article proposes a new face-swapping framework that includes ShapeEditor and a pretrained StyleGAN model. The pretrained model gives the proposed framework the potential to generate clear and realistic faces. The ShapeEditor encoder effectively extracts and integrates the attribute and identity information of the input images, then accurately maps them onto the W+ space, thus controlling StyleGAN to output the appropriate results. Extensive experiments show that the proposed method performs better than existing frameworks in terms of clarity and authenticity, with sufficiently integrating identity and attribute.

## Data Availability Statement

The original contributions presented in the study are included in the article/supplementary material, further inquiries can be directed to the corresponding author.

## Author Contributions

SY is responsible for code writing and thesis writing. KQ, RQ, PX, and SS are responsible for the inspiration of ideas. NL, LW, JC, GH, and BY put forward their opinions on the revision of the paper. All authors contributed to the article and approved the submitted version.

## Conflict of Interest

The authors declare that the research was conducted in the absence of any commercial or financial relationships that could be construed as a potential conflict of interest.

## Publisher's Note

All claims expressed in this article are solely those of the authors and do not necessarily represent those of their affiliated organizations, or those of the publisher, the editors and the reviewers. Any product that may be evaluated in this article, or claim that may be made by its manufacturer, is not guaranteed or endorsed by the publisher.
